# An Innovative Approach to Using Electronic Health Records Through Health Information Exchange to Build a Chronic Disease Registry in Michigan

**DOI:** 10.5888/pcd21.230413

**Published:** 2024-06-06

**Authors:** Olivia Barth, Beth Anderson, Kayla Jones, Adrienne Nickles, Kristina Dawkins, Akia Burnett, Krystal Quartermus

**Affiliations:** 1 Michigan Department of Health and Human Services, Lansing, Michigan; 2 Council of State and Territorial Epidemiologists Applied Public Health Informatics Fellowship Program, Atlanta, Georgia

## Abstract

Michigan’s CHRONICLE, the Chronic Disease Registry Linking Electronic Health Record Data, is a near–real-time disease monitoring system designed to harness electronic health record (EHR) data and existing health information exchange (HIE) infrastructure for transformative public health surveillance. Strong evidence indicates that using EHR data in chronic disease monitoring will provide rapid insight over time on health care use, outcomes, and public health interventions. We examined the potential of EHR data for chronic disease surveillance through close collaboration with our statewide HIE network and 2 participating health systems. We describe the development of CHRONICLE, the promising findings from its implementation, the identified challenges, and how those challenges will inform the next steps in testing, refining, and expanding the system. By detailing our approach to developing CHRONICLE and the considerations and early steps required to build an innovative, EHR-based chronic disease registry, we aim to inform public health leaders and professionals on the value of EHR data for chronic disease surveillance. With systematic testing, evaluation, and enhancement, our goal for CHRONICLE, as a fully realized and comprehensive surveillance system, is to model how collaborative health information exchange can support evidence-based strategies, resource allocation, and precision in disease monitoring.

**Summary**

**What is already known on this topic?**

Strategies for successful prevention and management of chronic disease are predicated on timely, detailed, and accurate data. Limitations exist in the chronic disease data systems used currently in public health practice, requiring data modernization efforts to address the rising burden and costs associated with chronic diseases.

**What is added by this report?**

Michigan has leveraged the state’s health information exchange (HIE) infrastructure and robust network of clinical data for longitudinal monitoring of hypertension and stroke. We examined information derived from hospital electronic health record data as a tool for rapid and actionable surveillance.

**What are the implications for public health practice?**

Clinical data shared across HIEs offer opportunities for innovative surveillance approaches and public health data enhancements.

## Background

### Chronic disease burden and surveillance limitations

Public health efforts and advances in medicine and technology have demonstrated success in preventing chronic disease and increasing life expectancy, but chronic conditions continue to be the leading cause of illnesses and deaths (1). In 2022, more than 65,000 deaths in Michigan could be attributed to chronic diseases (2). In 2021 chronic disease was the leading driver of national health care costs (3). We recognize that chronic disease prevalence and health disparities are likely the result of systemic differences in access to health care and treatment among particular demographic groups, such as Black, Hispanic, and Indigenous people; LGBTQIA+ people; and communities of low socioeconomic status (4,5). Public health practitioners are tasked with the critical responsibility of understanding and disseminating information on chronic disease prevalence, mortality, and disparities.

The limitations of existing disease surveillance systems are well-documented. They include a lack of time-critical access to data for dissemination of knowledge to prevent negative health outcomes, imperfect tools and data needed to address health inequities, and limited flexibility to accommodate rapid detection of emergent population health threats (6). Limitations also result from inadequate funding or resources to support robust data systems, data silos, fragmentation of surveillance systems, and a lack of coordination between health care and public health to enable data exchange. The COVID-19 pandemic reinforced the need for a modernized data infrastructure that can support effective response to emerging threats, drive systems change, and meaningfully address health equity (7). The value of informatics and a desire for more coordinated data systems that leverage clinical data to improve public health practice have long been recognized (6,8). Improving public health’s disease monitoring tools with the use of electronic health record (EHR) data is a transformative solution over classic surveillance approaches. Building modern and sustainable surveillance tools that use clinical data and address current and future needs requires coordination across all levels of public health. To do this, public health requires strategic investment and innovation in the surveillance tools and data necessary for continued success.

### Modernizing public health data

Recommendations from the Centers for Disease Control and Prevention’s Data Modernization Initiative can serve as foundational guidance (9). In 2022, Michigan released the *Michigan Health IT Roadmap “Bridge to Better Health” Report*, a guiding framework outlining the state’s most recent health information technology priorities (10). The report emphasizes the need for investment in statewide and local public health information technology infrastructure and data systems to support population health, emergency preparedness, and disease management capacity. Building public health systems that harness the abundant clinical data contained in EHRs and health information exchange (HIE) infrastructure is the next step in modernization.

In comparison to legacy public health data systems, EHR-based disease surveillance systems can offer more detailed information, timely access, and greater accuracy over self-reported data (11). The volume of EHR data makes it possible to examine population health trends at a more granular level, providing novel insights that can support community-led public health initiatives. With the adoption of EHRs and data sharing across clinical settings, HIE networks were established throughout the US to provide all members of a patient’s care team access to their clinical data to improve the quality of care and drive costs down (12). HIEs serve as a backbone for clinical data exchange (13) and offer a wealth of resources. Through strategic coordination across HIEs, clinical partners, and public health agencies, using EHR data and HIEs supports case investigation, public health alerts, linkage to care, and automated disease reporting (14,15). A handful of documented EHR-based public health surveillance systems, such as MENDS and NYC Macroscope (16,17), offer promising results for generating EHR-based chronic disease estimates (18,19). However, few existing EHR-based disease monitoring examples use an HIE network of clinical data for chronic disease surveillance.

In 2021, Michigan began building a chronic disease registry to address chronic disease and improve our understanding of the prevalence of these conditions. Michigan’s CHRONICLE (Chronic Disease Registry Linking Electronic Health Record Data) is a near–real-time statewide disease surveillance system designed to capture EHR data and explore chronic disease events throughout Michigan, beginning with stroke and hypertension. CHRONICLE is described as a near–real-time data system because it relies on event-driven data that are processed in real time but may be subject to data input or network delays. By collecting this information, we aim to improve longitudinal chronic disease surveillance capacity. CHRONICLE will provide timely chronic disease data with more granular detail and enable patient-level data linkage across disparate data systems for a more comprehensive disease monitoring ecosystem. We describe the development of CHRONICLE, a novel chronic disease surveillance tool introduced by the Michigan Department of Health and Human Services (MDHHS), that uses admission, discharge, and transfer (ADT) data to identify patients with a hypertension or stroke diagnosis and monitor disease burden longitudinally. Future enhancements to CHRONICLE will incorporate additional chronic conditions, data sources, and functionalities to enable more detailed examination of disease trends at a more granular geographic level, investigation of disease progression and comorbidities across the life course, and opportunities for innovative data linkages.

## System Development and Implementation

### Exploring data sources and models

**Figure 1:**
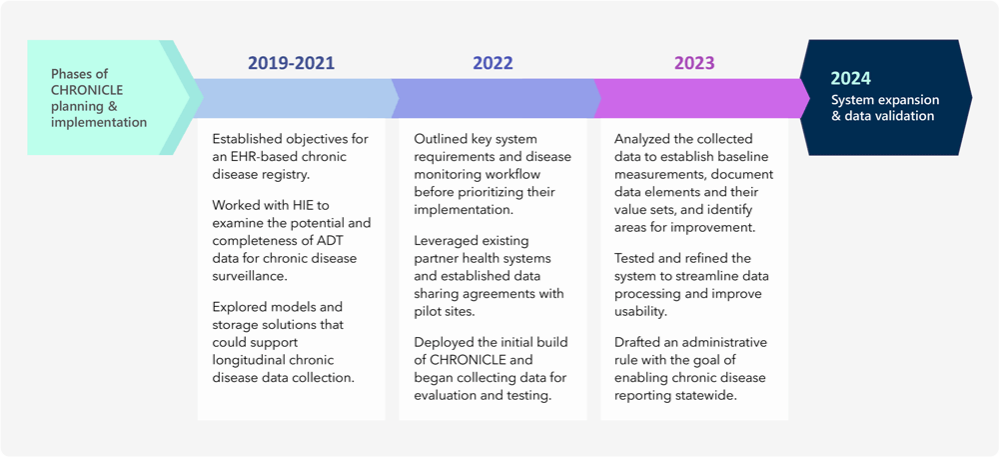
The phases of CHRONICLE’s (Chronic Disease Registry Linking Electronic Health Record Data) planning and implementation, from 2019 through 2023. Abbreviations: ADT, admission discharge transfer; EHR, electronic health record; HIE, health information exchange.

We examined various options for collecting clinical data that would effectively support longitudinal storage and patient-level data, advance data system capacity to drive improvement of disease prevention and management, and encourage innovative use of the data for public health cases. Michigan benefits from the presence of a centralized HIE, the Michigan Health Information Network (MiHIN), which maintains a broad reach across health care providers and settings. MiHIN reports that the network facilitates data exchange across 148 hospitals, 665 outpatient sites, 298 skilled nursing facilities, and 44 physician organizations, representing over 13 million patients (20). In 2019, we began working with MiHIN to explore the health information data shared across their network of sites and how those data could be translated to reliable surveillance estimates for chronic diseases (Figure 1).

MiHIN employs ADT notifications to support care coordination. ADTs are a Health Level 7 (HL7) (21) standard designed to minimize gaps in care by reporting alerts of changes to a patient’s health record to all members of that patient’s care team. ADTs contain information regarding health care visits in message segments, such as information on where the alert originated, who the patient is, and what observations or diagnoses have been made. Between 2019 and 2021, we monitored incoming hospital ADTs to better understand the completeness and quality of the data and the types and volume of ADT notifications to inform our system’s requirements. Variations in coding formats and value sets were observed across sites, as well as remarks on missingness of data fields across ADT message types (eg, admission versus discharge); however, the information contained in the ADTs demonstrated a promising opportunity for disease surveillance.

A goal in developing CHRONICLE was to ensure that the design of the system aligned with the MDHHS data strategy objectives to reduce further siloing of information and redundancies (10). While exploring potential models for the design of a longitudinal, rapid, chronic disease surveillance system, MDHHS was simultaneously assembling a statewide surveillance system to monitor the developing opioid crisis. The drug poisoning surveillance system, MiCelerity, was introduced in 2019 to collect drug poisoning events from ADTs and substance use–related deaths from the Electronic Death Registration System (22). It was built to national standards to enable secure data sharing and demonstrated a successful model that could be replicated for collecting chronic disease information from ADTs.

### Designing CHRONICLE and identifying partners

Before developing CHRONICLE, we identified key system requirements. The criteria for ADTs routed to CHRONICLE from the HIE include those with International Classification of Diseases (ICD)-10-CM codes (23) listed in the DG1 (diagnosis) segment of the ADT that contain at least 1 qualifying diagnosis code for hypertension or stroke (Table). The ADT message types identified for routing to CHRONICLE from the HIE are those indicating that a patient’s record was updated at admission, discharge, or registration. Next, we outlined the parameters for patient deduplication and linkage across visits to create a history of each patient’s chronic conditions. The system would create a unique patient identifier based on matching and scoring between the patient’s name, date of birth, sex, residence street name, and zip code, with automatic deduplication when scoring reached a particular threshold.

**Table:** Hypertension and Stroke ICD-10-CM Codes Selected for Inclusion in CHRONICLE and Used for Routing ADT Messages From MiHIN to CHRONICLE

ICD-10-CM code or series	Condition category	Description
**Hypertension**		
I10	Hypertension	Essential (primary) hypertension
I11	Hypertension	Hypertensive heart disease
I12	Hypertension	Hypertensive chronic kidney disease
I13	Hypertension	Hypertensive heart and chronic kidney disease
I15	Hypertension	Secondary hypertension
I16	Hypertensive crisis	Hypertensive crisis
I27	Pulmonary hypertension	Pulmonary hypertension
I97.3	Incidental elevated blood pressure	Postprocedural hypertension
O10	Pre-existing hypertension complicating pregnancy	Pre-existing hypertension complicating pregnancy
O11	Pre-existing hypertension complicating pregnancy	Pre-existing hypertension complicating pregnancy
O13	Gestational hypertension	Gestational hypertension
O16	Unspecified maternal hypertension	Unspecified maternal hypertension
R03.0	Elevated blood pressure	Elevated blood pressure reading, without diagnosis of hypertension
**Stroke**		
I60	Hemorrhagic stroke	Subarachnoid hemorrhage
I61	Hemorrhagic stroke	Intracerebral hemorrhage
I62	Hemorrhagic stroke	Other and unspecified intracranial hemorrhage
I63	Ischemic stroke	Cerebral infarction
I64	Unspecified stroke	Stroke, not specified as hemorrhage or infarction
I65	Ischemic stroke	Occlusion and stenosis of precerebral arteries, not resulting in cerebral infarction
I66	Ischemic stroke	Occlusion and stenosis of cerebral arteries, not resulting in cerebral infarction
I67	Other cerebrovascular disease	Other cerebrovascular diseases
I68	Cerebrovascular disorder in disease classified elsewhere	Cerebrovascular disorders in diseases classified elsewhere
I69	Sequelae of cerebrovascular disease	Sequelae of cerebrovascular disease

Abbreviations: ADT, admission, discharge, transfer; CHRONICLE, Chronic Disease Registry Linking Electronic Health Record Data; ICD, International Classification of Diseases (23); MiHIN, Michigan Health Information Network.

The second set of requirements concentrated on the user-facing functionalities and desired system outputs. Users with access to patient-level data could explore the patient listing pages and search for patients or events. Depending on a user’s role, reports at a patient or aggregate level could be exported for further analysis. Another requirement was that the system have a built-in dashboard that would showcase recent trends and general patient or event counts of the collected data. By leveraging MiCelerity’s existing ADT data feed, database infrastructure, and system security for CHRONICLE, we could satisfy numerous initial requirements before adapting the system to chronic disease monitoring.

In selecting hospital sites, we relied on our long-standing relationships with stroke centers participating in the Michigan Stroke Program (24). We selected 6 hospitals in 2 health systems located across mid- and southeast Michigan to pilot ADT data collection in CHRONICLE through their connection with MiHIN. Data sharing agreements were established with the health systems and HIE, outlining the ICD-10-CM codes that would flag the ADTs originating from participating hospitals for routing from MiHIN to CHRONICLE. Success in onboarding sites was a result of the existing relationships with the hospitals and clear communication of CHRONICLE’s aim to use the data to improve public health surveillance, health care, and patient outcomes. Another compelling argument for hospital participation was that their staff would not be burdened with extra work to submit data because of ongoing data sharing through the HIE.

### System testing and addressing data quality

**Figure 2:**
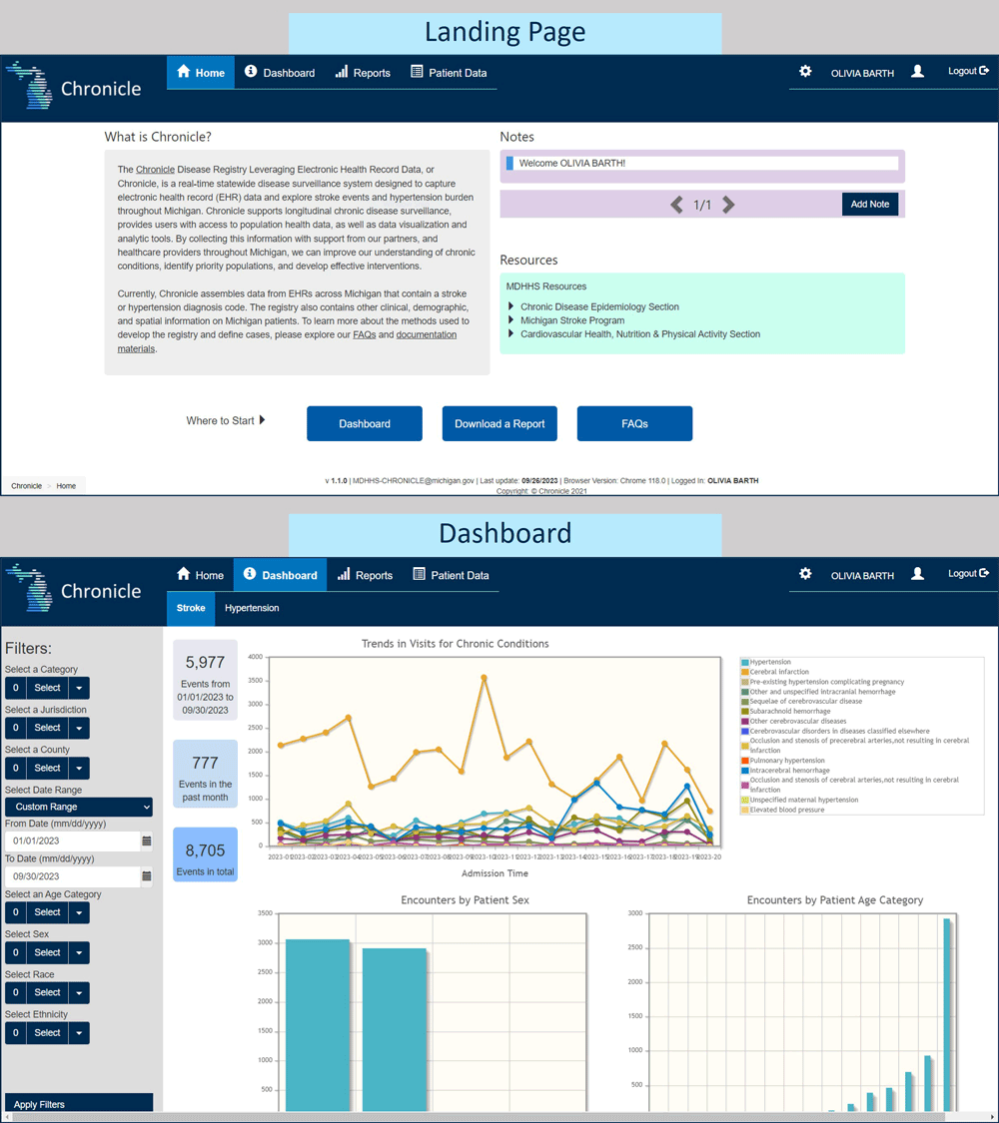
An archived landing page and dashboard view in CHRONICLE (Chronic Disease Registry Linking Electronic Health Record Data), a near–real-time disease monitoring system designed to harness electronic health record data and existing health information exchange infrastructure for transformative public health surveillance. Source: Michigan Department of Health and Human Services.

By September 2022, the first version of CHRONICLE was completed and began collecting an average of 800 ADT messages per day from participating sites. The system features a patient and event viewer and a dashboard to describe general trends in the data collected (Figure 2). In the first few months of monitoring incoming data, we addressed performance issues, updated the deduplication process to ensure proper functioning, and reviewed code sets across sites for fields such as race and ethnicity. Identification of such issues was the result of user story review and usability testing by the MDHHS staff. A recent finding suggested further enhancements are required to accurately capture information related to acute events. CHRONICLE will be updated with the introduction of event types, integration of each diagnosis code’s date of diagnosis, and adding diagnosis status (admitting, working, or final). Accounting for event types in CHRONICLE will aid in developing reliable disease estimates and electronic (e)-phenotypes, or computable definitions that use clinical data to specify a particular cohort. These and other findings are the result of regular monitoring, user testing, and evaluation of several dimensions of data quality, including data element agreement, comparison to HL7 definitions, and data completeness.

Additional investigation into CHRONICLE’s data quality has involved direct coordination with the hospitals to verify patient records against the information we have collected. Though these concordance analyses have been limited to a small sample of patients, they have highlighted the potential discrepancies that can exist between hospital EHR source data and ADT data. Currently, CHRONICLE is only accessible to the MDHHS staff. Once the ADT data has been validated and e-phenotypes established, our focus will shift to onboarding users, incorporating additional enhancements, and disseminating disease estimates and emerging trends data.

## Implications and Next Steps

### Lessons learned

The outset of CHRONICLE’s development dates back nearly 5 years, yet the system continues to be improved through testing, data validation, and adjusting methods to best fit the complex challenges encountered. Creating a clear roadmap for using EHR data in disease monitoring is critical to fostering effective partnerships and organizing scalable and innovative strategies. Before engaging in clinical data surveillance using HIE data, it is important to understand the HIE landscape. Examining HIE capacity for supporting public health strategies and documenting their current use cases (defined scenarios or processes that describe the purpose, specifications, rules, and requirements that enable health information exchange) may assist in identifying resources and key partners that will strengthen your approach. Developing CHRONICLE required a comprehensive assessment of the segments and fields in ADT data, their unique value sets and coding systems, level of missingness or variation in use, differences across facilities, and other questions that required collaboration with the HIE and health systems. For CHRONICLE, it was crucial to align with ongoing data modernization strategies (10) and demonstrate the potential of EHR data in public health.

### Challenges

Using EHR data is necessary for advancing public health, but it is not without serious limitations. Because of the nature of CHRONICLE as a secondary analysis tool for ADT data and the navigation of a complex data exchange landscape, implementation presents challenges. We recognize that there is still a critical lack of EHR data quality standards specific to public health use (25) and that dedicated efforts at the national, state, and regional levels are required to solidify broad use of EHR data in public health. We plan to support strategies that promote the establishment and reinforcement of such standards as we continue to document our findings. Strategies that foster data quality improvement will be important in leveraging HIE data for successful disease monitoring (26).

We recognize the limitations in our approach and the data to satisfy our long-term objectives. Currently, a gap exists in the literature on the use of ADT and HIE data in disease surveillance. This gap presents challenges in evaluating the data and developing validated disease measures. Beyond data quality concerns, ADT data are limited in what they contain, lacking information for exploring complex questions about disease progression, control, and severity. For hypertension, the lack of blood pressure measurements prevents the opportunity to examine the undiagnosed population. ADT data also pose an issue for generalizability of certain conditions because they are limited to patients seeking health care and may be biased toward more severe cases. Regular monitoring of ADT data is necessary to identify abnormal findings or delays in data processing that may affect data quality. We are currently documenting our findings on the quality of ADT data and continuing discussions with MiHIN, the health systems, and our ADT data users on methods to address these concerns. An ideal chronic disease surveillance system would include additional information from the hospital setting, such as laboratory values and vital signs along with other important clinical data on prescriptions and treatments. As a result, we plan to examine other clinical data sources and tools, such as continuity of care documents or Fast Healthcare Interoperability Resources (27), that could be used to supplement the ADT data in CHRONICLE.

Another consideration with the secondary use of clinical data for public health disease monitoring is potential legal barriers (28). In Michigan, we chose to leverage the ADT data network of our statewide HIE, which presented its own challenges because of the existing legal framework for data exchange under which the state and participating health care facilities operate. As a result, we established data sharing agreements with MiHIN and our pilot hospitals to enable data collection in CHRONICLE. This approach is not feasible in scaling the system statewide, and chronic diseases do not benefit from the reporting requirements defined for communicable diseases in Michigan’s Public Health Code. We explored several mechanisms to overcome data sharing barriers and determined that a statewide solution will require mandated chronic disease reporting through the institution of an administrative rule, as with other disease and injury reporting rules already in place (29,30). This approach avoids establishing agreements with each facility or provider organization and ensures scalable, statewide chronic disease monitoring in CHRONICLE across a variety of clinical settings. A priority in expanding CHRONICLE as a vital public health tool is exploring the unique data sharing challenges across other types of settings, such as community-based organizations and tribal health clinics. We want to ensure CHRONICLE is representative of the entire Michigan population and that data remain accessible to those trying to improve the health of our population.

### Future opportunities

As we continue to test CHRONICLE and collect data, plans have been set for its expansion and enhancements. Further investigation is necessary to determine the validity of hypertension and stroke estimates derived from ADT data, requiring comparison to gold standard data sources and methodology review for testing e-phenotypes. Additionally, a priority is to engage our statewide HIE, contributing pilot hospitals, payers, and other users of ADT data in activities focused on improving data usefulness through conformance and standardization. Future plans include broadening of the system’s user roles, improving CHRONICLE’s dashboard feature, building technical connections to enable linkage across MDHHS data systems, incorporating prehospital emergency medical service data, and integrating additional chronic conditions beyond stroke and hypertension. Additional diseases include asthma, diabetes, heart disease, high cholesterol, chronic kidney disease, and sickle cell disease. These enhancements will be a transformative step toward building CHRONICLE into a comprehensive chronic disease data system, enabling better disease and comorbidity monitoring across an array of linked data sources. Additionally, a focus will be placed on onboarding additional clinical sites and on broadening our partnerships to explore community information exchange and how to incorporate data on social determinants of health (31). These activities contribute to the enhancement of the system and represent important steps in demonstrating the merits of CHRONICLE as a disease monitoring tool.

### Conclusion

Improving disease monitoring systems to support chronic disease prevention and management and promotion of health equity is a priority in Michigan and has driven the development of CHRONICLE. Implementation of the system has followed a phased approach with the first iteration of CHRONICLE limited to a handful of data sources and chronic conditions. We envision the future system to be equipped with an extensive data repository and advanced functionality that will support evidence-based strategies and interventions to improve health outcomes and care.

## References

[1] Remington PL , Brownson RC ; Centers for Disease Control and Prevention. Fifty years of progress in chronic disease epidemiology and control. *MMWR Suppl.* 2011;60(4):70–77. 21976169

[2] Division for Vital Records & Health Statistics, Michigan Department of Health & Human Services. Number of Deaths and Age-adjusted Mortality Rates for the Ten Leading Causes of Death, Michigan and United States Residents, 2022. Updated December 19, 2023. Accessed March 18, 2024. https://vitalstats.michigan.gov/osr/chi/Deaths/leadUS/LeadingUSObject2.asp?AreaCode=0&AreaType=S&JS=No

[3] Centers for Disease Control and Prevention, National Center for Chronic Disease Prevention and Health Promotion. COVID-19 and Chronic Disease Prevention and Interventions. March 15, 2023. Accessed October 13, 2023. https://www.cdc.gov/chronicdisease/programs-impact/pop/covid-19.htm#

[4] Krieger N . Theories for social epidemiology in the 21st century: an ecosocial perspective. *Int J Epidemiol.* 2001;30(4):668–677. 10.1093/ije/30.4.668 11511581

[5] Warne D , Lajimodiere D . American Indian health disparities: psychosocial influences. *Soc Personal Psychol Compass.* 2015;9(10):567–579. 10.1111/spc3.12198

[6] Choi BC . The past, present, and future of public health surveillance. *Scientifica (Cairo).* 2012;2012:875253. . Erratum in: Scientifica (Cairo);2012;2012:6943062 10.6064/2012/875253 24278752 PMC3820481

[7] Lane JT , Smith K , Allen M , Surio P , Ruebush E . COVID-19 highlights critical need for public health data modernization to remain a priority. *J Public Health Manag Pract.* 2020;26(6):634–636. 10.1097/PHH.0000000000001268 32969954

[8] Groseclose SL , Buckeridge DL . Public health surveillance systems: recent advances in their use and evaluation. *Annu Rev Public Health.* 2017;38(1):57–79. 10.1146/annurev-publhealth-031816-044348 27992726

[9] Centers for Disease Control and Prevention. Data modernization initiative strategic implementation plan. December 22, 2021. Accessed October 13, 2023. https://www.cdc.gov/surveillance/data-modernization/priorities/index.html

[10] Michigan Department of Health and Human Services. Michigan health IT roadmap. “Bridge to Better Health” report. June 2022. Accessed October 13, 2023. https://www.michigan.gov/mdhhs/-/media/Project/Websites/mdhhs/Doing-Business-with-MDHHS/Boards-and-Commissions/Health-Information-Technology-Commission/CY2022-Bridge-to-Better-Health-Report_Adopted_Final-Aug22.pdf?rev=4dd6bf50a4d24d71a049c15f7032b524

[11] Vogel J , Brown JS , Land T , Platt R , Klompas M . MDPHnet: secure, distributed sharing of electronic health record data for public health surveillance, evaluation, and planning. *Am J Public Health.* 2014;104(12):2265–2270. 10.2105/AJPH.2014.302103 25322301 PMC4232140

[12] Walker J , Pan E , Johnston D , Adler-Milstein J , Bates DW , Middleton B . The value of health care information exchange and interoperability. *Health Aff (Millwood).* 2005;Suppl Web Exclusives:W5-10–W5-18. 10.1377/hlthaff.W5.10 15659453

[13] Kierkegaard P , Kaushal R , Vest JR . Applications of health information exchange information to public health practice. *AMIA Annu Symp Proc.* 2014;2014:795–804. 25954386 PMC4419901

[14] Shapiro JS , Mostashari F , Hripcsak G , Soulakis N , Kuperman G . Using health information exchange to improve public health. *Am J Public Health.* 2011;101(4):616–623. 10.2105/AJPH.2008.158980 21330598 PMC3052326

[15] Dixon BE , Grannis SJ , McAndrews C , Broyles AA , Mikels-Carrasco W , Wiensch A , . Leveraging data visualization and a statewide health information exchange to support COVID-19 surveillance and response: Application of public health informatics. *J Am Med Inform Assoc.* 2021;28(7):1363–1373. 10.1093/jamia/ocab004 33480419 PMC7928924

[16] Hohman KH , Martinez AK , Klompas M , Kraus EM , Li W , Carton TW , . Leveraging electronic health record data for timely chronic disease surveillance: the multi-state EHR-based network for disease surveillance. *J Public Health Manag Pract.* 2023;29(2):162–173. 10.1097/PHH.0000000000001693 36715594 PMC9897452

[17] Perlman SE , McVeigh KH , Thorpe LE , Jacobson L , Greene CM , Gwynn RC . Innovations in population health surveillance: using electronic health records for chronic disease surveillance. *Am J Public Health.* 2017;107(6):853–857. 10.2105/AJPH.2017.303813 28426302 PMC5425902

[18] Rassen JA , Bartels DB , Schneeweiss S , Patrick AR , Murk W . Measuring prevalence and incidence of chronic conditions in claims and electronic health record databases. *Clin Epidemiol.* 2018;11:1–15. 10.2147/CLEP.S181242 30588119 PMC6301730

[19] Hohman KH , Zambarano B , Klompas M , Wall HK , Kraus EM , Carton TW , . Development of a hypertension electronic phenotype for chronic disease surveillance in electronic health records: key analytic decisions and their effects. *Prev Chronic Dis.* 2023;20:E80. 10.5888/pcd20.230026 37708339 PMC10516201

[20] Michigan Health Information Network Shared Services. About Michigan Health Information Network. 2023. Accessed October 18, 2023. https://mihin.org/who-we-are-v22/

[21] HL7 International. Introduction to HL7 Standards. Accessed March 18, 2024. https://www.hl7.org/implement/standards/

[22] Michigan Department of Health and Human Services. MiCelerity – Frequently asked questions. April 2023. Accessed October 13, 2023. https://www.michigan.gov/mdhhs/-/media/Project/Websites/mdhhs/Keeping-Michigan-Healthy/Communicable-and-Chronic-Diseases/Michigan-Disease-Surveillance-System/MiCelerity_FAQs_V3.pdf?rev=6b1d7294442b48558565fe0beaaeb3d1&hash=BABEDDDC54D63F164FCF186385887376

[23] World Health Organization. International Classification of Diseases 11th Revision. Accessed March 18, 2024. https://icd.who.int/en

[24] Michigan Department of Health and Human Services. Michigan Stroke Program. 2023. Accessed October 18, 2023. https://www.michigan.gov/mdhhs/keep-mi-healthy/communicablediseases/epidemiology/chronicepi/stroke

[25] Lewis AE , Weiskopf N , Abrams ZB , Foraker R , Lai AM , Payne PRO , . Electronic health record data quality assessment and tools: a systematic review. *J Am Med Inform Assoc.* 2023;30(10):1730–1740. 10.1093/jamia/ocad120 37390812 PMC10531113

[26] Horth RZ , Wagstaff S , Jeppson T , Patel V , McClellan J , Bissonette N , . Use of electronic health records from a statewide health information exchange to support public health surveillance of diabetes and hypertension. *BMC Public Health.* 2019;19(1):1106. 10.1186/s12889-019-7367-z 31412826 PMC6694493

[27] Infrastructure FHIR . HL7 International. Updated January 31, 2022. Accessed March 18, 2024. https://www.hl7.org/Special/committees/fiwg/overview.cfm

[28] van Panhuis WG , Paul P , Emerson C , Grefenstette J , Wilder R , Herbst AJ , . A systematic review of barriers to data sharing in public health. *BMC Public Health.* 2014;14(1):1144. 10.1186/1471-2458-14-1144 25377061 PMC4239377

[29] Michigan Administrative Rules. Reporting of poisonings due to use of prescription or illicit drugs. 1978 PA 312, MCL 325.76 to 325.79. Accessed October 30, 2023. https://ars.apps.lara.state.mi.us/AdminCode/DownloadAdminCodeFile?FileName=R%20325.76%20to%20R%20325.79.pdf&ReturnHTML=True

[30] Michigan Department of Health and Human Services. Cancer Reporting. 1978 PA 312, MCL 325.9050 to 325.9057. Accessed October 30, 2023. https://ars.apps.lara.state.mi.us/AdminCode/DownloadAdminCodeFile?FileName=1577_2015-070HS_AdminCode.pdf&ReturnHTML=True

[31] Cantor MN , Thorpe L . Integrating data on social determinants of health into electronic health records. *Health Aff (Millwood).* 2018;37(4):585–590. 10.1377/hlthaff.2017.1252 29608369 PMC10995852

